# Reconsidering throat swab culture in melioidosis: diagnostic stewardship with clinical nuance

**DOI:** 10.1128/jcm.00809-26

**Published:** 2026-07-20

**Authors:** Nitin Gupta

**Affiliations:** 1 Department of Infectious Disease, Kasturba Medical College, Manipal Academy of Higher Education, Manipal, Karnataka, India; Mayo Clinic, Rochester, Minnesota, USA

**Keywords:** throat swab, diagnostic stewardship, melioidosis

## LETTER

I read with great interest the article by Duguid and colleagues (1). The authors should be congratulated for addressing an important and pragmatic question in melioidosis (1). Their large data set clearly demonstrates that routine throat and rectal swab culture in all patients with suspected melioidosis generates substantial laboratory workload, with low overall positivity. However, I would like to highlight a few contextual issues before these findings are extrapolated to other endemic settings.

First, the yield of throat swabs should be interpreted in relation to the limited sensitivity of other available diagnostic specimens, particularly blood culture. In suspected melioidosis, blood culture positivity is often far from universal, even in clinically significant disease (2). Moreover, in many endemic regions, especially outside tertiary-care centers, access to automated blood culture systems, adequate blood culture volumes, selective media, and reliable species-level identification may be inconsistent. Therefore, a specimen with modest sensitivity may still add diagnostic value when it is highly specific, easy to obtain, minimally invasive, and complementary to blood, sputum, pus, urine, or tissue culture. In the study by Duguid et al., throat swabs were positive in 25.3% of confirmed cases and in 36.0% of patients with pneumonia. Although throat swabs were rarely the sole positive specimen, the small proportion of exclusive diagnoses may still be clinically relevant in a disease where delayed microbiological confirmation can influence treatment decisions (1).

Second, the non-invasive nature of throat swab collection deserves emphasis. A throat swab is simple, inexpensive, repeatable, and generally acceptable across age groups. It may be particularly useful when sputum is unavailable, in children, or in patients with severe disease in whom invasive sampling is delayed or not feasible. From a clinical perspective, the value of a diagnostic sample is determined not only by its absolute positivity rate but also by feasibility, safety, specificity, and its ability to add incremental yield to the diagnostic bundle.

Third, the performance of throat swabs may vary by clinical syndrome and geography. The Darwin cohort is uniquely valuable, but the clinical spectrum of melioidosis differs across endemic regions. In parts of South and Southeast Asia, head-and-neck disease, including suppurative parotitis and cervical lymphadenitis, appears to be more frequently recognized than in northern Australia (3–5). In such settings, the upper aerodigestive tract may be more relevant for diagnosis, and throat swab yield may be higher than that observed in an Australian cohort (4). Therefore, diagnostic stewardship recommendations should ideally be syndrome-specific and region-specific rather than universally restrictive.

I agree with the authors that indiscriminate rectal swab culture in all patients with suspected melioidosis is unlikely to be efficient. However, for throat swabs, the conclusion may need to be more nuanced. Rather than abandoning routine use entirely, a targeted approach may be preferable: throat swab culture should be prioritized in suspected pulmonary melioidosis, in children, in patients unable to produce sputum, in patients with head-and-neck involvement, and in regions where parotid or upper airway-associated melioidosis is common.
